# Morel-Lavallée Lesion: Report of a Case of Unknown Mechanism

**DOI:** 10.1155/2015/947450

**Published:** 2015-02-17

**Authors:** Elissaios Kontis, Antonios Vezakis, Vasiliki Psychogiou, Panagiotis Kalogeropoulos, Andreas Polydorou, Georgios Fragulidis

**Affiliations:** 2nd Department of Surgery, Aretaieion Hospital, University of Athens, 76 Vas. Sofias Avenue, 115 28 Athens, Greece

## Abstract

Morel-Lavallée lesions are cystic lesions occurring between the subcutaneous tissue and the underlying layer of a fascia. The most frequent mechanism of occurrence is posttraumatic, usually after degloving injuries. The chain of events leading to the occurrence of this lesion is the formation of a potential space between the subcutaneous tissue and the firmly attached deep fascia, which in turn fills with blood and/or lymph and/or necrotic fat, secondary to disruption of the capillaries. We present a case of a 74-year-old male patient with a cystic lesion of the lateral surface of his left thigh increasing in size over a period of six months. Despite the meticulous history acquisition, we were unable to reveal any alleged mechanism of injury of the area. The patient underwent an MRI which revealed an ovoid cyst. The patient underwent surgical excision of the cyst. The pathology examination revealed a simple cyst, lined by a fibrous capsule and filled with serosanguineous fluid, thus confirming the diagnosis. The patient made a full recovery and since has been asymptomatic.

## 1. Introduction

Morel-Lavallée lesion refers to a benign cystic lesion occurring within a potential space between the subcutaneous tissue and the underlying fascia [1, 2]. Morel-Lavallée lesion represents a rare entity and has been associated with closed degloving injuries, secondary to blunt musculoskeletal injuries, usually over the greater trochanter but also in other locations such as the lower lumbar region, the thigh, or the calf [1, 3, 4]. The explanatory mechanism is that high-energy blunt injury exerts tangential shear force to the relatively mobile subcutaneous tissue, which in turn is “torn” away due to inertia from the firmly attached underlying fascia. This chain of events creates a potential space between the subcutaneous and the underlying fascia, which is filled with blood and/or lymph secondary to the disrupted perforating vessels/capillaries [1, 5].

## 2. Case Presentation

**Figure 1:**
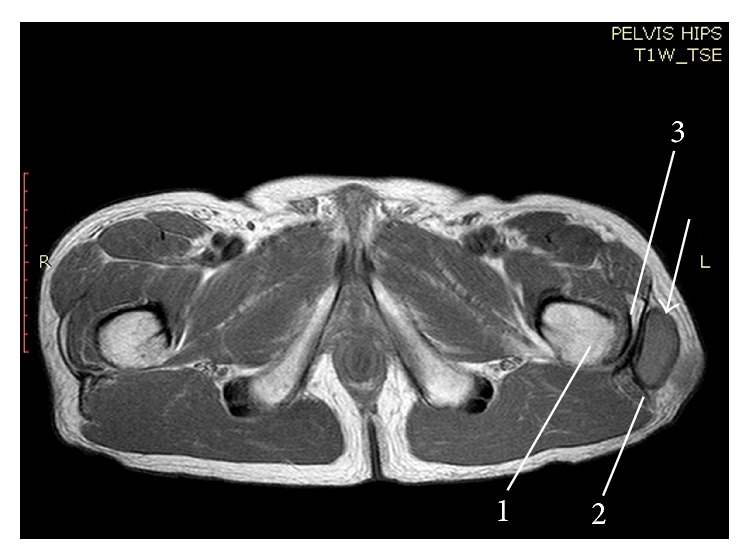
T1 weighted MRI image, depicting a cystic lesion (white arrow) located between the subcutaneous tissue and the underlying fascia of the iliotibial tract. There is no association of this lesion with the hip joint, findings consistent with a Morel-Lavallée lesion. (1) Greater trochanter, (2) iliotibial tract, and (3) the outer surface of the hip joint (L: left).

A 74-year-old male patient, with a past medical history of hypertension and chronic pain of his left hip joint, presented complaining of a cystic lump over his left hip joint which had increased in size during the past 6 months. The aforementioned lump was painless and was not associated with the movements of the hip joint. The patient did not report any recent medical history of injury of the associated area. Given his past medical history of chronic pain of his left hip joint, the patient underwent an MRI of the pelvis and thighs, in order to exclude a synovial cyst originating from the hip joint, secondary to chronic inflammation of the articular surfaces. The MRI revealed an ovoid cystic formation measuring 6.8 × 3.5 × 4.5 cm, with strong intensity signal on T1 and low on T2, lateral to the greater trochanter and the iliotibial tract (Figure 1). No pathological findings were found in the hip joint and the associated bony structures. Given the patient's discomfort and will to undergo surgical excision, he underwent surgical exploration and removal of this cystic formation. The pathological examination of the specimen revealed a cyst with fibrous wall, filled with dark brown fluid. The abovementioned findings were consistent with Morel-Lavallée lesion. The patient made a full recovery and since remains asymptomatic with no recurrence of the cyst.

## 3. Discussion

Morel-Lavallée lesion refers to a cystic lesion secondary to blunt injury and especially in degloving injuries. When tangential shear forces are exerted on the relatively mobile subcutaneous tissue, due to inertia, the subcutaneous tissue is torn away from the relatively firm underlying muscle fascia. This results in the creation of a potential space between the two associated planes, which in turn is filled with blood/and or lymph from the disrupted perforating vessels/capillaries [5]. Morel-Lavallée lesions are more often encountered over the trochanteric region or the proximal thigh; however it has been reported in other locations such as the lower lumbar region or the calf. Also they have been associated with pelvic or acetabular fractures [6]. Albeit, in a small fraction of patients, there is no mechanism of injury involved, only vigorous exercise of the anatomical area; thus some authors speculate that overuse or repeated microtrauma could result in a Morel-Lavallée lesion [3, 6]. However, we failed to elucidate a history of either trauma or overuse of the anatomic area with this patient.

The natural history of this lesion has not yet been established as it represents a rare entity. However some key steps of the process have been identified; after the initial formation of the potential blood filled space, there is evolution of this haematoma with absorption of the blood, which is replaced by serosanguineous fluid [1]. The last step in this chain of events is the formation of a peripheral fibrous capsule secondary to an anti-inflammatory reaction. The entrapment of fluid within the cyst may maintain a degree of chronic inflammation that could cause the gradual enlargement of the lesion over a long period of time. Most authors agree that the imaging modality of choice is MRI. However the findings of an MRI may vary significantly, based on the chronicity and internal contents of the lesion. Despite the rarity of this lesion, MRI imaging has been used for the only so far available classification system [7]. The differential diagnosis of a Morel-Lavallée lesion includes posttraumatic fat necrosis, coagulopathy-related hematoma, and posttraumatic early stage myositis ossificans [8]. The available treatment modalities depend on the timing of identification of a Morel-Lavallée lesion. If it is detected during the acute phase, percutaneous drainage and compression may be sufficient. However the establishment of the peripheral fibrous capsule of the lesion and entrapment of large volume of fluid (of more than 50 mL) renders drainage and conservative measures ineffective and is associated with increased recurrence risk, thus with surgical excision being the only effective modality for the long-standing Morel-Lavallée lesion [8, 9]. In conclusion, we presented the rare case of a Morel-Lavallée lesion of unknown cause. Although Morel-Lavallée lesions are usually associated with trauma, it should be included also in cystic lumps of the peripelvic area. MRI is the best imaging modality for characterizing Morel-Lavallée lesions. Surgical excision is the preferable treatment modality when the lesion is long standing or includes large volume of fluid.
